# Removal of Parotid Gland

**Published:** 1883-01

**Authors:** Claude Browning


ARTICLE IV.

### REMOVAL OF PAROTID GLAND.

REPORTED BY CLAUDE BROWNING, M. D.

(Hospital of Oral Surgery, Philadelphia, Clinic of Prof. Jas. E. Garretson, M. D.)

The patient, a professional gentleman residing in Colorado, came as a private patient to the hospital with a view to the extripation of a parotid tumor, the bulk and relations of which were such as to render life no longer bearable under its presence. The origin of the growth is referred to a sudden sharp crack occurring in the neighborhood of the ear, heard by the gentleman as some few years back he was sitting at the open window of a car while crossing the Rocky Mountains, which crack the operation showed to have as its meaning a fracture of the styloid process of the os temporis. Measurement of the morbid enlargement gave four and a half inches vertically, three transversely, and one and a half in thickness. Fixation amounted to almost entire immobility. Diagnosis: Fibro-enchondroma.

The operator, having the assistance of Professors Agnew and Brinton and of Dr. Hunt, of the Pennsylvania Hospital, commenced the ablation by a first incision extending from the temple to a position upon the neck overlying the space of selection for the ligation of the carotid externus. A second incision, extended transversely across the cheek, met the first adjacent to the anti-tragus of the ear. The incisions thus made affording two anterior and one great posterior flap, the tumor was uncovered, the cuts being made of a depth sufficient to expose the carotid, which vessel was lifted and tied. Attempt to dislodge the mass exhibited it as completely filling the zygomatic fossa, and at the same time inseparably connected with the periosteal structure of the maxillary ramus, both upon its external and deep faces, similar relations existing with the mastoid process of the os temporis; the styloid was found fractured.

The removal of the growth, which, after an hour's labor was effected by means of much dissection and careful manipulation (the latter particularly demanded on account of insinuation of prolongations of the tumor into the spleno-maxillary fossa), was secured in a manner that admitted of no doubt as to a radical extirpation. After securing the carotid artery not a single ligature was used, the facial having been tied at the time of uncovering.

At the time of operation Professor Garretson remarked that all tumors of this region, and the removal of them as well, involve markedly the lymphatic system, hence a change to be expected lies in the direction of lymphatic stasis. In the case of the present ablation the process of local healing progressed entirely satisfactorily, but after about a month an expression of coma began to show itself, which continued to increase until later the patient died in Chicago. Sectio cadaveris revealed the cause of death to lie in an abscess situated at the base of the brain.

